# Supplementary material for: Investigating the impact of the years of blindness on sleep rhythms, dream patterns, and spatial abilities: The BLINDREAM protocol
Source: PLoS One. 2025 Jul 2;20(7):e0327521. doi: 10.1371/journal.pone.0327521 (PMC12221037; doi:10.1371/journal.pone.0327521)
Supplement: Attachment — Submitted filename: PONE-D-25-01572_reviewer.pdf [file pone.0327521.s001.pdf]

PLOS ONE

Investigating the Impact of the Years of Blindness on Sleep Rhythms, Dream Patterns, and Spatial Abilities: The BLINDREAM Protocol

--Manuscript Draft--

**Manuscript Number:**	PONE-D-25-01572
**Article Type:**	Study Protocol
**Full Title:**	Investigating the Impact of the Years of Blindness on Sleep Rhythms, Dream Patterns, and Spatial Abilities: The BLINDREAM Protocol
**Short Title:**	The BLINDREAM Protocol
**Corresponding Author:**	Helene Vitali<br>Istituto Italiano di Tecnologia<br>Genova, GE ITALY
**Keywords:**	blindness; sleep; circadian; dream; spatial processing
**Abstract:**	Sleep health is a topic of great interest in recent years. However, it is still often undervalued in vulnerable populations, such as those with sensory disorders. A higher percentage of blind individuals experience circadian disorders, and their dreams have different sensory contents. However, many questions remain unanswered regarding the impact of blindness on overall sleep structure and how circadian desynchronization may influence them. Additionally, understanding how changes in the dream sensory contents and sleep alterations may affect daily life skills, such as spatial cognition which tends to be more challenging in blind individuals, is relevant for further comprehension of the vision role on our perception. The BLINDREAM protocol aims to investigate the interrelationships of all these aspects. We aim to collect one week of data from 20 blind adults and 20 sighted age-matched controls. Participants' sleep and circadian rhythm will be assessed through one-night home polysomnography, melatonin sampling, one-week actigraphy monitoring, and questionnaires. Dream activity will be measured through a one-week voice-recorded dream diary and questionnaires. Finally, a neuropsychological assessment of spatial cognition will be conducted. Capturing the dynamics of subjective experiences phase-locked to neural and physiological evaluation both between and within individuals, this approach can contribute to our understanding of the impact of blindness on sleep processes.
**Order of Authors:**	Helene Vitali<br>Claudio Campus<br>Laura Giorgetti<br>Francesco Famà<br>Pietro Mattioli<br>Dario Arnaldi<br>Monica Gori
**Additional Information:**	
**Question**	**Response**
**Financial Disclosure**<br><br>Enter a financial disclosure statement that describes the sources of funding for the work included in this submission. Review the submission guidelines for detailed requirements. View published research articles from *PLOS ONE* for specific examples.	Yes

Powered by Editorial Manager® and ProduXion Manager® from Aries Systems Corporation

This statement is required for submission and **will appear in the published article** if the submission is accepted. Please make sure it is accurate.<br><br>**Funded studies**<br>Enter a statement with the following details:<br>• Initials of the authors who received each award<br>• Grant numbers awarded to each author<br>• The full name of each funder<br>• URL of each funder website<br>• Did the sponsors or funders play any role in the study design, data collection and analysis, decision to publish, or preparation of the manuscript?<br><br>Did you receive funding for this work?	
Please add funding details.<br>as follow-up to "**Financial Disclosure**<br><br>Enter a financial disclosure statement that describes the sources of funding for the work included in this submission. Review the submission guidelines for detailed requirements. View published research articles from *PLOS ONE* for specific examples.<br><br>This statement is required for submission and **will appear in the published article** if the submission is accepted. Please make sure it is accurate.<br><br>**Funded studies**<br>Enter a statement with the following details:<br>• Initials of the authors who received each award<br>• Grant numbers awarded to each author<br>• The full name of each funder<br>• URL of each funder website<br>• Did the sponsors or funders play any role in the study design, data collection and analysis, decision to publish, or preparation of the manuscript?	This research was partially supported by the European Research Council (PI Monica Gori; Grant agreement No. 948349).<br>https://erc.europa.eu/homepage<br>No the funder plays any role in the study design, data collection and analysis, decision to publish, or preparation of the manuscript.

Did you receive funding for this work?"	
Please select the country of your main research funder (please select carefully as in some cases this is used in fee calculation).<br>as follow-up to "**Financial Disclosure**<br><br>Enter a financial disclosure statement that describes the sources of funding for the work included in this submission. Review the submission guidelines for detailed requirements. View published research articles from *PLOS ONE* for specific examples.<br><br>This statement is required for submission and **will appear in the published article** if the submission is accepted. Please make sure it is accurate.<br><br>**Funded studies**<br>Enter a statement with the following details:<br>• Initials of the authors who received each award<br>• Grant numbers awarded to each author<br>• The full name of each funder<br>• URL of each funder website<br>• Did the sponsors or funders play any role in the study design, data collection and analysis, decision to publish, or preparation of the manuscript?<br><br>Did you receive funding for this work?"	ITALY - IT
**Competing Interests**<br><br>Use the instructions below to enter a competing interest statement for this submission. On behalf of all authors, disclose any competing interests that could be perceived to bias this work—acknowledging all financial support and any other relevant financial or non-financial competing interests.<br><br>This statement is required for submission	NO authors have competing interests<br>Enter: The authors have declared that no competing interests exist.

and **will appear in the published article** if the submission is accepted. Please make sure it is accurate and that any funding sources listed in your Funding Information later in the submission form are also declared in your Financial Disclosure statement.<br><br>View published research articles from *PLOS ONE* for specific examples.<br><br>**NO authors have competing interests**<br><br>Enter: *The authors have declared that no competing interests exist.*<br><br>**Authors with competing interests**<br><br>Enter competing interest details beginning with this statement:<br><br>*I have read the journal's policy and the authors of this manuscript have the following competing interests: [insert competing interests here]*<br><br>* typeset	
**Ethics Statement**<br><br>Enter an ethics statement for this submission. This statement is required if the study involved:<br><br>• Human participants<br>• Human specimens or tissue<br>• Vertebrate animals or cephalopods<br>• Vertebrate embryos or tissues<br>• Field research<br><br>Write "N/A" if the submission does not require an ethics statement.<br><br>General guidance is provided below. Consult the submission guidelines for detailed instructions. **Make sure that all information entered here is included in the Methods section of the manuscript.**	This study, titled "The impact of years of blindness on sleep and dream processes and the relationship with spatial abilities", was approved by the Liguria Regional Ethics Committee (623/2022 - DB id 12600) and registered on ClinicalTrials.gov (ID NCT06631807).<br>written consent will be obtained for each participant

**Format for specific study types**<br><br>**Human Subject Research (involving human participants and/or tissue)**<br>• Give the name of the institutional review board or ethics committee that approved the study<br>• Include the approval number and/or a statement indicating approval of this research<br>• Indicate the form of consent obtained (written/oral) or the reason that consent was not obtained (e.g. the data were analyzed anonymously)<br><br>**Animal Research (involving vertebrate animals, embryos or tissues)**<br>• Provide the name of the Institutional Animal Care and Use Committee (IACUC) or other relevant ethics board that reviewed the study protocol, and indicate whether they approved this research or granted a formal waiver of ethical approval<br>• Include an approval number if one was obtained<br>• If the study involved *non-human primates*, add *additional details* about animal welfare and steps taken to ameliorate suffering<br>• If anesthesia, euthanasia, or any kind of animal sacrifice is part of the study, include briefly which substances and/or methods were applied<br><br>**Field Research**<br><br>Include the following details if this study involves the collection of plant, animal, or other materials from a natural setting:<br>• Field permit number<br>• Name of the institution or relevant body that granted permission	
**Data Availability**<br><br>Authors are required to make the data underlying their research findings fully available, without restriction.<br><br>If the manuscript reports pilot or preliminary results, the data underlying those results must be made available at the time of publication. A Data Availability	N/A - No results are reported

Powered by Editorial Manager® and ProduXion Manager® from Aries Systems Corporation

Statement describing where the data can be found is required at submission. Your answers to this question constitute the Data Availability Statement and will be published in the article, if accepted. When legal or ethical requirements prohibit public data sharing, authors must indicate how researchers may obtain access to the data. See the PLOS Data Policy and FAQ for detailed information.<br><br>**Important:** Stating 'data available on request from the author' is not sufficient. If your data are only available upon request, select 'No' for the first question and explain your situation in the text box.<br><br>For protocols without pilot or preliminary data, authors are strongly encouraged to state how they plan to share research data from their study when it is completed or published.<br><br>If your study includes pilot data, will they be made fully available without restriction?	
**Describe where the data may be found in full sentences. If you are copying our sample text, replace any instances of XXX with the appropriate details.**<br><br>• If **no pilot data are reported** in the manuscript, please enter the following: *No datasets were generated or analysed during the current study. All relevant data from this study will be made available upon study completion.*<br>• If the manuscript reports pilot data **held in a public repository**, include URLs, accession numbers or DOIs. If this information will only be available after acceptance, indicate this by ticking the box below. For example: *All XXX files are available from the XXX database (accession number(s) XXX, XXX.).*<br>• If the manuscript reports pilot data contained **within the manuscript and/or**	No datasets were generated or analysed during the current study. All relevant data from this study will be made available upon study completion.

**Supporting Information files**, enter the following:<br>*All relevant data are within the manuscript and its Supporting Information files.*<br>• If neither of these applies but you are able to provide **details of access elsewhere**, with or without limitations, please do so. For example:<br><br>*Data cannot be shared publicly because of [XXX]. Data are available from the XXX Institutional Data Access / Ethics Committee (contact via XXX) for researchers who meet the criteria for access to confidential data.*<br><br>*The data underlying the results presented in the study are available from (include the name of the third party and contact information or URL).*<br>• This text is appropriate if the data are owned by a third party and authors do not have permission to share the data.<br><br>For study protocols with data management or sharing plans, authors are encouraged to briefly describe in their Data Availability Statement how data they generate will be made accessible when the study is completed. For example:<br>• Deidentified research data will be made publicly available when the study is completed and published.<br><br>* typeset	
Additional data availability information:	

Powered by Editorial Manager® and ProduXion Manager® from Aries Systems Corporation

Manuscript

Click here to access/download;Manuscript;main_BLINDREAM_protocol.pdf

Investigating the Impact of the Years of Blindness on Sleep Rhythms, Dream Patterns, and Spatial Abilities: The BLINDREAM Protocol

Helene Vitali[1], Claudio Campus[1], Laura Giorgetti[2,3], Francesco Famà[2,3], Pietro Mattioli[2,3], Dario Arnaldi[2,3,†], Monica Gori[1,†]

[1]Unit for Visually Impaired People, Istituto Italiano di Tecnologia, 16152, Genova, Italy
[2]Clinical Neurophysiology, IRCCS Ospedale Policlinico San Martino, 16132, Genova, Italy.
[3]DINOGMI, University of Genova, Genova, Italy.

* Corresponding author: Helene.Vitali@iit.it, Fondazione Istituto Italiano di Tecnologia, Via E. Melen, 83 - 16152 Genova (Italy)
[†] These authors contributed equally to this work

ERC

Abstract

Sleep health is a topic of great interest in recent years. However, it is still often undervalued in vulnerable populations, such as those with sensory disorders. A higher percentage of blind individuals experience circadian disorders, and their dreams have different sensory contents. However, many questions remain unanswered regarding the impact of blindness on overall sleep structure and how circadian desynchronization may influence them. Additionally, understanding how changes in the dream sensory contents and sleep alterations may affect daily life skills, such as spatial cognition which tends to be more challenging in blind individuals, is relevant for further comprehension of the vision role on our perception. The BLINDREAM protocol aims to investigate the interrelationships of all these aspects. We aim to collect one week of data from 20 blind adults and 20 sighted age-matched controls. Participants' sleep and circadian rhythm will be assessed through one-night home polysomnography, melatonin sampling, one-week actigraphy monitoring, and questionnaires. Dream activity will be measured through a one-week voice-recorded dream diary and questionnaires. Finally, a neuropsychological assessment of spatial cognition will be conducted. Capturing the dynamics of subjective experiences phase-locked to neural and physiological evaluation both between and within individuals, this approach can contribute to our understanding of the impact of blindness on sleep processes.

Keywords: blindness, sleep, circadian, dream, spatial processing

Introduction

The study of sleep has gained increasing attention in recent years due to increased awareness among the general population. However, its relationship to sensory processing and how sleep processes are affected in sensory disabilities still remain intriguing questions. Blindness, in particular, is a great model to study the role of vision on sleep processes. The lack of vision poses unique challenges to sleep regulation, primarily due to the disruption of the circadian process, which is regulated by the light-dark cycle [1]. This disruption can lead to sleep phase disorders, most notably Non-24-Hour Sleep-Wake Disorder (N24SWD), a condition that affects approximately 72.2% of blind individuals [2,3]. N24SWD results from a misalignment between the internal circadian clock and the 24-hour day, causing significant difficulties in maintaining regular sleep patterns and impacting daily life [4]. Despite the known association between vision loss and circadian rhythm disturbances, the relationship between the years of blindness and circadian desynchronization remains insufficiently studied. Furthermore, while melatonin is significantly influenced by light exposure, its secretion patterns in blind individuals vary widely, ranging from similar to sighted individuals to highly disorganized [5], depending on the degree of vision loss [6].

The circadian rhythm disruptions could also affect the organization of the sleep stages. The impact of blindness on these sleep stages is still a topic of debate, with some studies suggesting alterations in sleep stages and microstructure [7–11], potentially due to the plastic changes in brain structures induced by visual deprivation, while other studies report no significant changes [12,13]. However, it remains unclear whether these changes are a direct result of blindness or a consequence of circadian rhythm desynchronization [14]. Indeed, even in childhood, before circadian disorders typically manifest, blind children report more sleep complaints and already exhibit an alteration of the sleep macro and microstructure [13,15–21].

These disruptions not only affect sleep architecture but should also have a broader impact on daily life activity [22–24]. However, only one study has evaluated the effects of sleep phase disorders on alertness, mood, and task performance in blind adults [25]. Given the difficulties that blind individuals experience in spatial perception and cognition [26–34], and the role of sleep in processing and consolidating spatial information (e.g. Sawangjit et al., 2020; Simon et al., 2022; Walker & Stickgold, 2004; Wamsley et al., 2010; Wilson & McNaughton, 1994), it is possible that disruptions in sleep mechanisms could further exacerbate these challenges. Despite the strong connection between these aspects, nowadays, little is known about how blindness might influence the relationship between sleep, including dreams, and spatial cognition.

The content of dreams is also altered by blindness (for a review, see Ilic et al., 2023; Vitali et al., 2022). For example, visual content is typically absent in the dreams of congenitally blind individuals or those who lost their sight early in life [42–44], whereas auditory, tactile, gustatory, and olfactory elements are more prominent [43,45]. Interestingly, those with late-onset blindness who had some visual experience earlier in life report a higher percentage of visual content in their dreams, though still less than sighted individuals [42,43]. The relationship between the onset of blindness and the presence of visual dream content suggests an inverse correlation, with earlier onset leading to less visual imagery in dreams [42,43]. The impact of these different dream experiences on spatial cognitive functions, is an area of growing interest [41,45]. Can the varied processing of dream content influence how sensory information is processed during our waking experiences, significantly affecting long-term performance, such as spatial abilities?

This study presented with this protocol aims to address these gaps by investigating the impact of blindness on sleep processes, dream content, and their relationship with spatial perception and memory. By exploring the links between clinical, psychological, neurobiological, and electrophysiological measures, this research seeks to provide a comprehensive understanding of how blindness influences these interconnected domains.

Materials and methods

Participants

We are aiming to recruit 40 adults: 20 Blind/Severely Visually Impaired (BSI) individuals and 20 age- and biological sex-matched Sighted (S) controls.

Sample size

Given the proof-of-concept nature of the study and the unavailability of prior effect size estimates in the relationship between the different measures, calculating power is currently not feasible. Thus, the sample size is based on a provisional and ambitious estimate of recruitment capacity using the *pwr* package on the R environment [46]. However, previous literature studies compared between groups some of the measures that we are considering separately, such as sleep macrostructure [5,8,47,9,11,13,14,19,20,25,44], sleep microstructure [12], dream contents [42,43,45], spatial perception task [26–28,48], and spatial memory tasks [33,34]. A representative sample size for each category is showed following:

1. Sleep architecture and circadian: A previous study investigated the differences in the power spectral activity between early and late blind in the different sleep stages, considering

circadian desynchronization as a covariate. According to the power analysis for the ANCOVA, with $\alpha = 0.05$ and power = 0.80, a total of 22 participants (11 early blind and 11 late blind) are needed to achieve a significant result if a medium effect size (Cohen's f = 0.897) is expected, as indicated by the generalized eta squared ($\eta g^2 = 0.446$) to detect differences in delta activity in the N3 stage [12].

2. Dream visual contents: A study investigated the significant differences in the percentage of dreams with visual content between congenital blind (CB), late blind (LB), and sighted controls (SC). Based on a Kruskal-Wallis test with $\eta^2 = 0.429$, $\alpha=0.05$, and desired power = 0.80, a total sample size of 18 participants (6 for each group) is required across the three groups (CB, LB, SC) to detect a significant difference in visual dream content [43].
3. Spatial perception task: The perception task that we will perform during this protocol study was previously evaluated between early blind and sighted controls [48]. According to the power analysis for the two-way ANOVA, with $\alpha = 0.05$ and power = 0.80, a total of 18 participants (9 blind and 9 sighted) are needed to achieve a significant result if a medium effect size (Cohen's f = 0.577) is expected, as indicated by the generalized eta squared ($\eta g^2 = 0.25$) to detect a difference in the spatial perception task.
4. Spatial memory task: The memory task we will perform during this study was previously evaluated between participants with visual deficits and sighted controls [34]. According to the power analysis for the ANOVA, with $\alpha = 0.05$ and power = 0.80, a total of 13 participants (6 blind and 7 sighted) are needed to achieve a significant result if a large effect size (Cohen's f = 0.85) is expected, as indicated by the generalized eta squared ($\eta g^2 = 0.419$) to detect a difference in the memory span.

Based on these statistical analyses of the sample size, a number of 10 blind and 10 sighted participants should be sufficient to detect significant and reliable differences between groups on different variables. This agrees with most of the sleep studies in blind participants that showed a sample number of 11/12 for the experimental and control group [7,8,42,48,11,12,14,26–28,33,34]. However, given the interest in investigating the onset of blindness, the high risk of withdrawal among blind participants due to the complexity of the protocol, and the proof-of-concept nature of the study exploring the interrelationship between various measures, we provisionally considered a sample size of 40 participants (20 BSI and 20 S). Nonetheless, efforts will be made to conduct interim analyses to estimate effect sizes based on the primary outcome, allowing for adjustments to the initial assumptions as necessary.

Inclusion and exclusion criteria

Inclusion criteria for the S group also include normal or corrected-to-normal vision, while for the BSI group, an impairment of the peripheral visual system (i.e., involving pre-chiasmatic structures, such as the retina and optic nerve). The visual deficit can be congenital (from birth) or have a late onset. Participants with visual impairment, classified according to the current diagnostic criteria, must have residual vision lower than 1.3 LogMAR. Inclusion criteria for both groups of participants will be as follows: age between 18 and 85 years; any gender and sex; any ethnicity, provided they have a good knowledge of the Italian language. Participants will be excluded from the study if they meet any of the following conditions: Tactile hypersensitivity (specifically, the ability to tolerate the equipment will be assessed); Hearing impairments; Use or history of using neuroactive drugs or substances in the six months prior to the study; Clinically significant and uncontrolled medical conditions; Central nervous system disorders, including a history of seizures or convulsive episodes of any kind, even minor; Uncontrolled cardiopulmonary conditions that could affect sleep macro- or microstructure; Pregnant women. IQ scores below the normal threshold according to a recognized international scale.

Recruitment and Screening

The clinical investigation commenced with the enrollment of the first patient on April 4th, 2024, and the recruitment period is scheduled to conclude in October 2025. An extension of the recruitment period will be considered if the required number of participants is not reached. Potential participants have been informed about the study via the Italian Institute of Technology mailing list, to which they have previously consented to be registered, or through communications with collaboration centers. All participants have been recruited at the IRCCS Ospedale Policlinico San Martino. The control group individuals have been recruited following the recruitment of the first experimental group and have been selected based on their characteristics (biological sex and age). Participants have been reimbursed for expenses/lost earnings related to traveling to the study site at the end of the protocol, with a maximum value of 75 euros (divided into 3 phases corresponding to the number of times they need to reach the venue). Before initiating data collection, participants have received comprehensive study information and the consent form. Inclusion criteria have been evaluated verbally and by considering demographics during this screening stage. If all criteria have been met, participants have been deemed eligible for the study.

Criteria for Withdrawal/Discontinuation of Participation

The participant involvement in the study will end under the following conditions: 1) at their request to discontinue early; 2) if logistical or technical issues arise; 3) if they no longer meet the inclusion/exclusion criteria; 4) or at the discretion of the experimenter. Throughout the study, a

subject's continued participation is contingent upon their adherence to the inclusion and exclusion criteria. If these criteria change, the experimenter will reassess the subject's participation, and the study may be discontinued to ensure the subject's safety and well-being, as well as the integrity of the data. Data collected up until that point will be retained and analyzed. The participant may withdraw from participation at any time without being required to provide a justification.

Design and Procedure

This study, titled "The impact of years of blindness on sleep and dream processes and the relationship with spatial abilities", was approved by the Liguria Regional Ethics Committee (623/2022 - DB id 12600) on 11th March 2024 and registered on ClinicalTrials.gov (ID NCT06631807).

The study design involves three experimental phases, with a total duration of 7 or 8 days per participant, as shown in Figure 1. The protocol was designed for 8 days to avoid overwhelming the participant with tasks on the first night. However, if the participant finds it more comfortable and simpler, all the equipment provided in visit 2 will be already directly provided in visit 1, shortening the protocol to 7 days but without reducing the information collected.

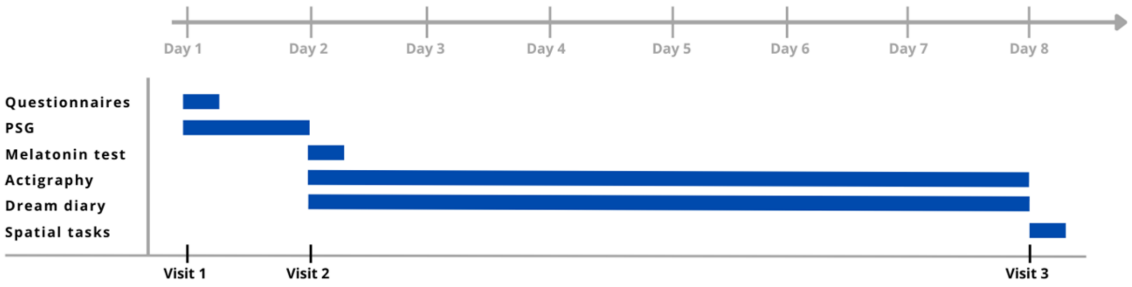

Figure 1. Study design

The three phases include three different visits at the IRCCS San Martino Hospital sleep lab, during which various measurements will be assessed. All the technologies used in the protocol are summarized in Figure 2.

Phase and Visit 1: Screening and sleep and dream assessment

The protocol will start verifying the inclusion and exclusion criteria (if not done during the recruiting phase). If the participant is eligible and agrees to take part in the study, written consent, and demographic data will be collected after reading the consent document to the participant, and the ~~sleep measurement~~ will start.

Visit 1 defines the t=0 of the protocol and includes the measurements of sleep and dream quality using questionnaires, and sleep quantity by home-polysomnography (PSG) for one night.

The questionnaires will evaluate sleep quality through the Pittsburgh Sleep Quality Index (PSQI) [49,50], Morningness-Eveningness Questionnaire (MEQ) [51], and Pre-Screening Questionnaire to Predict N24HSWD in Blind Subjects [52], and dream quality using the Dream Recall Frequency Scale (DRFS) [53], and Van Dream Anxiety Scale (VDAS) [54]. The experimenter will read the questionnaires and collect the answers. We will also collect information on the sensory content of their dreams using a customized questionnaire based on previous studies [42,43]. Full polysomnography will then be set up using home polysomnograph Compumedics Somtè PSG2, conducted according to current international standards of the AASM [55], with the following channels: EEG (F3, F4, C3, C4, O1, O2), two monopolar EOG channels, three submental EMG channels (two recording electrodes and one reference), nasal cannula for oronasal airflow, peripheral pulse oximeter, thoraco-abdominal movement bands, and tibialis anterior EMG. Once it is set up, the participant will remain seated for a few minutes at rest, or they may be asked to perform tasks to measure background brain activity, such as closing and opening the eyes. Afterward, they will be discharged with the home polysomnograph and instructed to return the following day to remove the device.

Phase and Visit 2: Circadian and dream assessment.

Visit 2 will take place the day after Visit 1. The phase 2 will begin with the removal of the polysomnograph. Afterward, the participant will be fitted with an actigraphy bracelet, which they must wear for the following week. The actigraphy recording (Act-Trust Condor Instrument) records the body movement, skin and ambient temperature, and ambient light.

On the first day of recording, ~~at~~ the participants will be asked to collect five consecutive saliva samples, one hour apart in the evening, at home, for melatonin concentration analysis (Sleep Check Kit; Buhlmann). The samples will be collected independently by the participant, at one-hour intervals, so that the fourth sample (t=0) coincides with their typical bedtime (-3, -2, -1, 0, and +1). The five test tubes for sample collection will be provided with a number of rubber bands on the edge corresponding to the number of the test tube to be considered. Therefore, at timepoint 1, test tube n 1 will have one rubber band; at timepoint 2, test tube n 2 will have two rubber bands, and so on. This has proven to be a simple and effective method for blind people to recognize the correct test tube. The participant will also be asked to keep a dream diary throughout the week. For this, they will be asked to verbally record information about the dream activity from the previous night.

Phase and Visit 3: Neuropsychological assessment.

Visit 3 will be done one week after providing the actigraphic bracelet and recorder. This phase evaluates the spatial perceptual and mnemonic functions in a session lasting approximately two hours maximum. The tests will be assessed at the same time of the day in all participants to avoid

circadian variations. The neuropsychological tests will include the spatiotemporal acoustic bisection task [48] and the audio-corsi test (Setti et al., 2018). In the first task, participants will be asked to judge the spatial or temporal relationship between three acoustic stimuli provided by an array of speakers (as shown in Figure 2). For spatial memory assessment, the experiments will use the equipment for the audio-corsi test (Setti et al., 2018, 2023). The participant will listen to a series of white noise sounds moving along a predefined path in space. The participant's task will be to remember the spatial sequence of the different sounds. In both tests, the stimuli are controlled by a laptop with dedicated Matlab software that allows us to define stimulation parameters and record responses.

A.

Phase	Technology	Model	Assessment
1	PSG	Compumedics Somtè PSG2	Sleep architecture and microstructure
2	Actigraphy	Act-Trust Condor Instrument	Circadian rhythm
2	Melatonin	Sleep Check Kit; Buhlmann	Circadian rhythm
2	Recorder	TASCAM DR-05 Linear	Dream diary
3	Speakers	-	Spatiotemporal audio bisection task
3	Audio-Corsi	-	Audio-Corsi task

B.

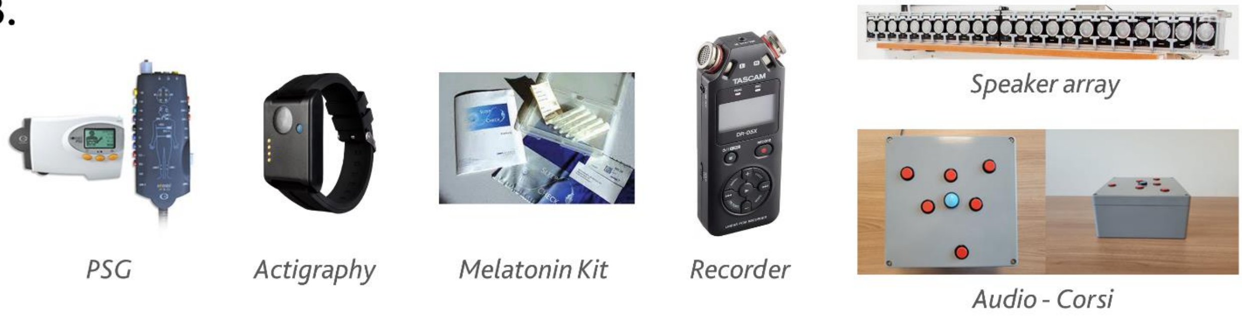

Figure 2. Technologies used in the protocol study. 2.A. Table of technologies used for each phase. 2.B. picture of the technologies.

Data Management Plan

Data management will be conducted in accordance with the General Data Protection Regulation (GDPR) and with the requirements of the Declarations of Helsinki [57]. Each participant will be assigned a general alphanumerical code (e.g., S001) at the beginning of the protocol. Personal-

identifiable data will be kept separately from the experimental data, and the code to decrypt the personal data will only be available to authorized personnel.

Experimental data will be collected using a paper-based Case Report Form (CRF) in a pseudonymized format, which will be subsequently transferred to the Foundation for entry into a secure database hosted on a protected cloud platform. This database will be accessible exclusively to authorized personnel, with a tracking of all modifications. Security measures are robust, ensuring that pseudonymized data will be stored in duplicates in the institute's internal repository, thereby limiting access to authorized staff only. To further bolster data protection, comprehensive training on data protection principles will be provided to all staff involved, alongside established protocols for managing potential data breaches. Informed consent and collected data obtained from each participant will be securely archived for ten years, ensuring that access to personal data remains restricted to authorized staff.

Data Analysis Plan

Hypotheses

Given that the primary sleep disorder in blind individuals is the N24SWD (S. Aubin et al., 2016; Dirks et al., 2019), depending on the degree of vision loss (Lockley et al., 1997) and that the circadian misalignment could alter daily life performance [22–25]. The first hypothesis aims to investigate these aspects. Specifically, H1.1. would investigate whether visual deprivation causes a desynchronization of the circadian rhythm in congenitally BSI individuals and those who experience late-onset blindness after many years of visual deprivation. Previous evidence shows that blind people with a late onset but many years of blindness are more likely to resemble the permitted blind in their performance [48]. H1.2. would explore if greater circadian desynchronization correlates with poorer performance on spatial perception and memory tasks in blind individuals.

The circadian rhythm disruptions could also affect the sleep architecture. However, it is not completely clear whether the alterations in sleep quality, architecture, and microstructure in blind individuals are due to plastic changes in brain structures induced by visual deprivation or only to circadian misalignment [7–14]. Therefore, H2.1. would investigate whether circadian desynchronization affects sleep structure in BSI individuals and whether this is not the only factor influencing sleep structure. If the circadian rhythm is not the only factor, H2.2. would investigate whether a correlation exists between sleep macro and microstructure and spatial performance. Indeed, we hypothesize that even BSI participants with a normally modulated circadian rhythm by

the light-dark cycle may exhibit significant differences compared to controls, involving sleep patterns related to sensorimotor processing and the consolidation of spatial information.
Finally, it was reported that the sensory content of dreams is also affected by blindness (for a review, see Ilic et al., 2023; Vitali et al., 2022), with visual dream content inversely correlated with the onset of blindness [42,43]. Given the impact of dream experiences on spatial cognitive functions [41,45], H3.1. would explore whether there is a significant relationship between the frequency of dream recall and spatial tasks in both groups. Specifically, we hypothesize that a higher frequency of dream recall predicts better spatial abilities. Moreover, H3.2. would investigate whether the sensory dream contents can influence spatial abilities, hypothesizing that a greater presence of visual dream content predicts better performance. We also hypothesize that a higher frequency of eye movement may predict better spatial skills in blind individuals.

Statistical Analysis

Data analysis for investigating all hypotheses will involve parametric and non-parametric tests, with group differences assessed using t-tests, ANOVA, and linear or generalized linear mixed models as applicable. Both between-group and within-group correlations are of interest. In cases of statistical significance, an appropriate post-hoc test will be performed. Where necessary, results will be corrected for multiple comparisons. A significance level of $p < 0.05$ will be applied. Standard software will include MATLAB and R [46], which are widely recognized in research contexts. The EEGlab[58], FieldTrip [59], and custom Matlab scripts will be used to analyze electroencephalographic data.
Specifically, H1.1 and H1.2 will be tested using data from actigraphy and melatonin samples correlated to clinical data and performance in the audio bisection and audio-corsi tasks. The circadian data will be analyzed to determine potential shift phases and circadian disorders.
For the sleep quality and quantity analysis and their relationship with circadian rhythm (H2), we will also consider PSG data and questionnaires (PSQI, MEQ, Pre-Screening for N24HSWD). Sleep scoring will be performed based on PSG data. Then, from EEG data, we will analyze both sleep macrostructure (statistics and spectrogram) and sleep microstructure (spindles and slow waves). For H2.1. the circadian phase will be considered a covariate in the models, as performed in previous articles [12,14]. Control and BSI groups will also be compared for H2.1. and H2.2.
Finally, for the assessment of dream role (H3), the correlation between dream questionnaires (DRF, VDAS, dream sensory contents scale) and spatial performance will be evaluated considering both within and between group analysis. Further analysis will also consider one-week dream reports to extract the frequency of dream sensory contents as previously performed [45]. The eye movement activity will be extracted from EOG data during PSG [42].

Data Availability

The datasets that will be generated from this study will be shared online in Zenodo repository.

Discussion and Limitations

This study addresses the relationship between different aspects of sleep and spatial performance in blind individuals. Indeed, little is known about how blindness might influence the relationship between sleep, including dreams, and spatial cognition. Could circadian disruption be the primary factor behind most of the sleep structure differences between blind and sighted individuals, or are other mechanisms involved? How does information reprocessing change compared to a person with typical vision, and how might this affect spatial performance? Is the key found in dreams or in neurophysiological biomarkers?

Sleep disruptions have a strong impact on daily life activity [22–24], but one study has evaluated the relationship between daily performance and sleep in blind adults [25]. An interesting aspect to explore could be the relationship between sleep and spatial abilities in blind individuals. Indeed, on one side, numerous studies have highlighted the role of sleep and dreams in processing and consolidating spatial information (e.g., Sawangjit et al., 2020; Simon et al., 2022; Walker & Stickgold, 2004; Wamsley et al., 2010; Wilson & McNaughton, 1994), and insufficient sleep impacts the ability to perceive the space around us [60]. On the other side, blind individuals often struggle with both perceptual and memory-based spatial tasks (Gori et al., 2014; Setti et al., 2018; 2021), particularly under high cognitive load conditions. Scientific research on multisensory integration shows that vision provides the most precise and reliable information about the spatial properties of the external world, thus having a dominant influence on spatial perception [61,62]. Indeed, vision offers an immediate and detailed view of the environment with high spatial resolution [63], making it crucial for aligning neural representations of space across different senses [62,64]. The dominance of vision in spatial reasoning becomes especially clear when it interacts with other senses. For instance, during sensory conflicts, auditory and tactile perceptions are strongly shaped by visual information presented at the same time [65–68]. Therefore, the absence of visual experience from birth alters the ability to perform complex spatial tasks [26–34]. However, most of these spatial difficulties do not manifest in late blind individuals [30,69–71], suggesting that the first years of life can constitute a sensitive period for the development of spatial metrics. Nevertheless, as the years of blindness increase, spatial challenges grow, becoming similar between early and late blind individuals after about 20 years [48]. Despite the significant connection between these factors, little is currently known about how blindness may impact the relationship

between sleep and spatial cognition. This study seeks to fill these gaps by examining the connections between clinical, psychological, neurobiological, and electrophysiological factors, offering a thorough understanding of how blindness impacts these interrelated areas. Moreover, the results could guide the development of new rehabilitative strategies or tools tailored to the needs of blind individuals, enhancing their quality of life and cognitive functioning. Indeed, different studies suggest that sensory stimulation during sleep, such as auditory or vibrotactile cues, may enhance the activation of newly learned memories, potentially improving spatial performance [72–74].

However, some limitations persist regarding sample size and the capacity to fully characterize visual impairment. Nonetheless, given the challenges of recruiting participants for a week-long observational study, significant findings may still emerge. We will try to account for the characterization of factors defining visual impairment. Additional limitations relate to the applicability of microstructural analyses with low-density EEG; nonetheless, we aim to apply more advanced analyses than those typically found in the literature. Despite these constraints, we believe the findings from this study will contribute substantial insights and advancements to research in this field.

Acknowledgment

This research was partially supported by the European Research Council (PI Monica Gori; Grant agreement No. 948349).

References

1. Hull JT, Czeisler CA, Lockley SW. Suppression of Melatonin Secretion in Totally Visually Blind People by Ocular Exposure to White Light: Clinical Characteristics. Ophthalmology. 2018. doi:10.1016/j.ophtha.2018.01.036
2. Aubin S, Gacon C, Jennum P, Ptito M, Kupers R. Altered sleep–wake patterns in blindness: a combined actigraphy and psychometric study. Sleep Med. 2016. doi:10.1016/j.sleep.2016.07.021
3. Dirks C, Grünewald D, Young P, Heidbreder A. Pilot study to investigate sleep disorders in the blind and persons with relevant visual impairment. Ophthalmologe. 2019. doi:10.1007/s00347-018-0723-z
4. Hartley S, Dauvilliers Y, Quera-Salva MA. Circadian Rhythm Disturbances in the Blind. Curr Neurol Neurosci Rep. 2018;18: 1–8. doi:10.1007/s11910-018-0876-9
5. Lewy AJ, Newsome DA. Different types of melatonin circadian secretory rhythms in some

blind subjects. J Clin Endocrinol Metab. 1983. doi:10.1210/jcem-56-6-1103

6. Lockley SW, Skene DJ, Arendt J, Tabandeh H, Bird AC, Defrance R. Relationship between melatonin rhythms and visual loss in the blind. J Clin Endocrinol Metab. 1997. doi:10.1210/jc.82.11.3763

7. Ayala-guerrero F, Mexicano G. Sleep Characteristics in Blind Subjects. J Sleep Disord Manag. 2015;1.

8. Hono T, Hiroshige Y, Miyata Y. Nocturnal sleep spindle activity in blind subjects. Psychiatry and Clinical Neurosciences. 2000. doi:10.1046/j.1440-1819.2000.00674.x

9. Hono T, Hiroshige Y, Miyata Y. A case report on EEG nocturnal sleep in visually impaired persons aged in their 30s and 50s. Psychiatry Clin Neurosci. 1999;53: 145–147. doi:10.1046/j.1440-1819.1999.00501.x

10. Scrofani A, Cioni M, Filetti S, Lanaia F, Pennisi G, Bella R, et al. Changes in sleep spindle activity of subjects with chronic somatosensitive and sensorial deficits. Preliminary results. Neurol Sci. 1996. doi:10.1007/bf01997717

11. Berger RJ, Olley P, Oswald I. The EEG, eye-movements and dreams of the blind. Q J Exp Psychol. 1962;14: 183–186. doi:10.1080/17470216208416534

12. Aubin S, Christensen JAE, Jennum P, Nielsen T, Kupers R, Ptito M. Preserved sleep microstructure in blind individuals. Sleep Med. 2018. doi:10.1016/j.sleep.2017.11.1135

13. Leger D, Guilleminault C, Santos C, Paillard M. Sleep/wake cycles in the dark: Sleep recorded by polysomnography in 26 totally blind subjects compared to controls. Clin Neurophysiol. 2002;113: 1607–1614. doi:10.1016/S1388-2457(02)00221-3

14. Aubin S, Jennum P, Nielsen T, Kupers R, Ptito M. Sleep structure in blindness is influenced by circadian desynchrony. J Sleep Res. 2018. doi:10.1111/jsr.12548

15. Adhikari S, van Nispen RMA, Poudel M, van Rens F, Elsman EBM, van der Werf YD, et al. Sleep Patterns in Children With Blindness: A Comparison With Normally Sighted Peers. Invest Ophthalmol Vis Sci. 2023;64: 46. doi:10.1167/iovs.64.14.46

16. Fazzi E, Zaccagnino M, Gahagan S, Capsoni C, Signorini S, Ariaudo G, et al. Sleep disturbances in visually impaired toddlers. Brain Dev. 2008;30: 572–578. doi:10.1016/j.braindev.2008.01.008

17. Hayton J, Marshall J, Dimitriou D. Lights Out: Examining Sleep in Children with Vision Impairment. Brain Sci. 2021;11. doi:10.3390/brainsci11040421

18. Ingram DG, Cruz JM, Stahl ED, Carr NM, Lind LJ, Keirns CC. Sleep Challenges and Interventions in Children With Visual Impairment. J Pediatr Ophthalmol \& Strabismus. 2022;59: 77–86. doi:10.3928/01913913-20210623-01

19. Leger D, Guilleminault C, Defrance R, Domont A, Paillard M. Prevalence of sleep/wake disorders in persons with blindness. Clin Sci. 1999;97: 193–199. doi:10.1042/cs0970193
20. Leger D, Stal V, Quera-Salva MA, Guilleminault C, Paillard M. Disorders of wakefulness and sleep in blind patients. Rev Neurol (Paris). 2001;157: S135-9.
21. Vitali H, Campus C, Signorini S, De Giorgis V, Morelli F, Varesio C, et al. Blindness affects the developmental trajectory of the sleeping brain. Neuroimage. 2024;286: 120508. doi:10.1016/j.neuroimage.2024.120508
22. Depner CM, Stothard ER, Wright KP. Metabolic consequences of sleep and circadian disorders. Curr Diab Rep. 2014. doi:10.1007/s11892-014-0507-z
23. Chellappa SL. Circadian misalignment: A biological basis for mood vulnerability in shift work. Eur J Neurosci. 2020. doi:10.1111/ejn.14871
24. Richardson C, Micic G, Cain N, Bartel K, Maddock B, Gradisar M. Cognitive performance in adolescents with Delayed Sleep-Wake Phase Disorder: Treatment effects and a comparison with good sleepers. J Adolesc. 2018. doi:10.1016/j.adolescence.2018.03.002
25. Lockley SW, Dijk DJ, Kosti O, Skene DJ, Arendt J. Alertness, mood and performance rhythm disturbances associated with circadian sleep disorders in the blind. J Sleep Res. 2008. doi:10.1111/j.1365-2869.2008.00656.x
26. Gori M, Sandini G, Martinoli C, Burr DC. Impairment of auditory spatial localization in congenitally blind human subjects. Brain. 2014;137: 288–293. doi:10.1093/brain/awt311
27. Kolarik AJ, Pardhan S, Cirstea S, Moore BCJ. Auditory spatial representations of the world are compressed in blind humans. Exp brain Res. 2017;235: 597–606. doi:10.1007/s00221-016-4823-1
28. Kolarik AJ, Cirstea S, Pardhan S. Evidence for enhanced discrimination of virtual auditory distance among blind listeners using level and direct-to-reverberant cues. Exp brain Res. 2013;224: 623–633. doi:10.1007/s00221-012-3340-0
29. Cattaneo Z, Vecchi T, Cornoldi C, Mammarella I, Bonino D, Ricciardi E, et al. Imagery and spatial processes in blindness and visual impairment. Neurosci Biobehav Rev. 2008;32: 1346–1360. doi:10.1016/j.neubiorev.2008.05.002
30. Cappagli G, Cocchi E, Gori M. Auditory and proprioceptive spatial impairments in blind children and adults. Dev Sci. 2017;20. doi:10.1111/desc.12374
31. Pasqualotto A, Proulx MJ. The role of visual experience for the neural basis of spatial cognition. Neurosci Biobehav Rev. 2012;36: 1179–1187. doi:10.1016/j.neubiorev.2012.01.008
32. Radziun D, Korczyk M, Szwed M, Ehrsson HH. Are blind individuals immune to bodily

illusions? Somatic rubber hand illusion in the blind revisited. Behav Brain Res. 2024;460: 114818. doi:10.1016/j.bbr.2023.114818

33. Setti W, Cuturi LF, Engel I, Picinali L, Gori M. The influence of early visual deprivation on audio-spatial working memory. Neuropsychology. 2021. doi:10.1037/neu0000776

34. Setti W, Cuturi LF, Cocchi E, Gori M. A novel paradigm to study spatial memory skills in blind individuals through the auditory modality. Sci Rep. 2018;8: 1–10. doi:10.1038/s41598-018-31588-y

35. Wilson MA, McNaughton BL. Reactivation of hippocampal ensemble memories during sleep. Science. 1994;265: 676–679. doi:10.1126/science.8036517

36. Walker MP, Stickgold R. Sleep-dependent learning and memory consolidation. Neuron. 2004;44: 121–133. doi:10.1016/j.neuron.2004.08.031

37. Simon KC, Clemenson GD, Zhang J, Sattari N, Shuster AE, Clayton B, et al. Sleep facilitates spatial memory but not navigation using the Minecraft Memory and Navigation task. Proc Natl Acad Sci U S A. 2022;119: e2202394119. doi:10.1073/pnas.2202394119

38. Sawangjit A, Oyanedel CN, Niethard N, Born J, Inostroza M. Deepened sleep makes hippocampal spatial memory more persistent. Neurobiol Learn Mem. 2020;173: 107245. doi:10.1016/j.nlm.2020.107245

39. Wamsley EJ, Tucker M, Payne JD, Benavides JA, Stickgold R. Dreaming of a Learning Task Is Associated with Enhanced Sleep-Dependent Memory Consolidation. Curr Biol. 2010;20: 850–855. doi:10.1016/j.cub.2010.03.027

40. Vitali H, Campus C, De Giorgis V, Signorini S, Gori M. The vision of dreams: from ontogeny to dream engineering in blindness. J Clin sleep Med JCSM Off Publ Am Acad Sleep Med. 2022;18: 2051–2062. doi:10.5664/jcsm.10026

41. Ilic K, Bertani R, Lapteva N, Drakatos P, Delogu A, Raheel K, et al. Visuo-spatial imagery in dreams of congenitally and early blind: a systematic review. Front Integr Neurosci. 2023;17: 1–11. doi:10.3389/fnint.2023.1204129

42. Christensen JAE, Aubin S, Nielsen T, Ptito M, Kupers R, Jennum P. Rapid eye movements are reduced in blind individuals. J Sleep Res. 2019. doi:10.1111/jsr.12866

43. Meaidi A, Jennum P, Ptito M, Kupers R. The sensory construction of dreams and nightmare frequency in congenitally blind and late blind individuals. Sleep Med. 2014. doi:10.1016/j.sleep.2013.12.008

44. Kerr NH, Foulkes D, Schmidt M. The structure of laboratory dream reports in blind and sighted subjects. J Nerv Ment Dis. 1982. doi:10.1097/00005053-198205000-00006

45. Kang J, Bertani R, Raheel K, Soteriou M, Rosenzweig J, Valentin A, et al. Mental Imagery

in Dreams of Congenitally Blind People. Brain Sci. 2023;13: 1–9. doi:10.3390/brainsci13101394

46. R Core Team. R: A Language and Environment for Statistical Computing. Vienna, Austria: R Foundation for Statistical Computing; 2024. Available: https://www.r-project.org

47. Lockley SW, Skene DJ, Butler LJ, Arendt J. Sleep and activity rhythms are related to circadian phase in the blind. Sleep. 1999;22: 616–623. doi:10.1093/sleep/22.5.616

48. Amadeo MB, Campus C, Gori M, Ossandon JP. Years of Blindness Lead to " Visualize " Space Through Time. 2020;14: 1–14. doi:10.3389/fnins.2020.00812

49. Mollayeva T, Thurairajah P, Burton K, Mollayeva S, Shapiro CM, Colantonio A. The Pittsburgh sleep quality index as a screening tool for sleep dysfunction in clinical and non-clinical samples: A systematic review and meta-analysis. Sleep Med Rev. 2016;25: 52–73. doi:10.1016/j.smrv.2015.01.009

50. Curcio G, Tempesta D, Scarlata S, Marzano C, Moroni F, Rossini PM, et al. Validity of the Italian version of the Pittsburgh Sleep Quality Index (PSQI). Neurol Sci Off J Ital Neurol Soc Ital Soc Clin Neurophysiol. 2013;34: 511–519. doi:10.1007/s10072-012-1085-y

51. Horne JA, Ostberg O. A self-assessment questionnaire to determine morningness-eveningness in human circadian rhythms. Int J Chronobiol. 1976;4: 97–110.

52. Flynn-Evans EE, Lockley SW. A Pre-Screening Questionnaire to Predict Non-24-Hour Sleep-Wake Rhythm Disorder (N24HSWD) among the Blind. J Clin sleep Med JCSM Off Publ Am Acad Sleep Med. 2016;12: 703–710. doi:10.5664/jcsm.5800

53. Schredl M. Reliability and stability of a dream recall frequency scale. Percept Mot Skills. 2004;98: 1422–1426. doi:10.2466/pms.98.3c.1422-1426

54. Ağargün MY, Kara H, Bilici M, Çilli AS, Telci M, Semiz ÜB, et al. The Van Dream Anxiety Scale: A subjective measure of dream anxiety in nightmare sufferers. Sleep Hypn. 1999;1: 204–211.

55. Berry R, Quan S, Abreu A, Bibbs M, DelRosso L, Harding S, et al. The AASM Manual for the Scoring of Sleep and Associated Events: Rules, Terminology and Technical Specifications. Version 2. Darien, IL: Darien, IL: America Accademy of Sleep Medicine; 2020.

56. Setti W, Vitali H, Campus C, Picinali L, Setti WMG. Audio-Corsi: A novel system to evaluate audio-spatial memory skills. Proc Annu Int Conf IEEE Eng Med Biol Soc EMBS. 2023; 3–6. doi:10.1109/EMBC40787.2023.10339960

57. World Medical Association. World Medical Association Declaration of Helsinki: ethical principles for medical research involving human subjects. JAMA. 2013;310: 2191–2194.

doi:10.1001/jama.2013.281053
58. Delorme A, Makeig S. EEGLAB: an open source toolbox for analysis of single-trial EEG dynamics including independent component analysis. J Neurosci Methods. 2004;134: 9–21. doi:10.1016/j.jneumeth.2003.10.009
59. Oostenveld R, Fries P, Maris E, Schoffelen J-M, Oostenveld R, Fries P, et al. FieldTrip: Open Source Software for Advanced Analysis of MEG, EEG, and Invasive Electrophysiological Data, FieldTrip: Open Source Software for Advanced Analysis of MEG, EEG, and Invasive Electrophysiological Data. Comput Intell Neurosci. 2011. doi:10.1155/2011/156869, 10.1155/2011/156869
60. Valera S, Guadagni V, Slone E, Burles F, Ferrara M, Campbell T, et al. Poor sleep quality affects spatial orientation in virtual environments. Sleep Sci (Sao Paulo, Brazil). 2016;9: 225–231. doi:10.1016/j.slsci.2016.10.005
61. Alais D, Burr D. The Ventriloquist Effect Results from Near-Optimal Bimodal Integration. Curr Biol. 2004;14: 257–262. doi:https://doi.org/10.1016/j.cub.2004.01.029
62. Welch RB, Warren DH. Immediate perceptual response to intersensory discrepancy. Psychol Bull. 1980;88: 638–667.
63. Tinti C, Adenzato M, Tamietto M, Cornoldi C. Visual experience is not necessary for efficient survey spatial cognition: evidence from blindness. Q J Exp Psychol (Hove). 2006;59: 1306–1328. doi:10.1080/17470210500214275
64. King AJ. What happens to your hearing if you are born blind? Brain. 2014;137: 6–8. doi:10.1093/brain/awt346
65. Anderson PW, Zahorik P. Auditory/visual distance estimation: accuracy and variability. Front Psychol. 2014;5: 1097. doi:10.3389/fpsyg.2014.01097
66. Bertelson P, Aschersleben G. Temporal ventriloquism: crossmodal interaction on the time dimension. 1. Evidence from auditory-visual temporal order judgment. Int J Psychophysiol Off J Int Organ Psychophysiol. 2003;50: 147–155. doi:10.1016/s0167-8760(03)00130-2
67. Botvinick M, Cohen J. Rubber hands ‘feel’ touch that eyes see. Nature. 1998;391: 756. doi:10.1038/35784
68. Flanagan JR, Beltzner MA. Independence of perceptual and sensorimotor predictions in the size-weight illusion. Nat Neurosci. 2000;3: 737–741. doi:10.1038/76701
69. Collignon O, Champoux F, Voss P, Lepore F. Sensory rehabilitation in the plastic brain. Prog Brain Res. 2011;191: 211–231. doi:10.1016/B978-0-444-53752-2.00003-5
70. Finocchietti S, Cappagli G, Gori M. Encoding audio motion: spatial impairment in early blind individuals. Front Psychol. 2015;6: 1357. doi:10.3389/fpsyg.2015.01357

71. Voss P, Gougoux F, Zatorre RJ, Lassonde M, Lepore F. Differential occipital responses in early- and late-blind individuals during a sound-source discrimination task. Neuroimage. 2008;40: 746–758. doi:10.1016/j.neuroimage.2007.12.020

72. Ngo H-V V, Martinetz T, Born J, Mölle M. Auditory closed-loop stimulation of the sleep slow oscillation enhances memory. Neuron. 2013;78: 545–553. doi:10.1016/j.neuron.2013.03.006

73. Shimizu RE, Connolly PM, Cellini N, Armstrong DM, Hernandez LT, Estrada R, et al. Closed-Loop Targeted Memory Reactivation during Sleep Improves Spatial Navigation. Front Hum Neurosci. 2018;12: 28. doi:10.3389/fnhum.2018.00028

74. Choi SH, Kwon H Bin, Jin HW, Yoon H, Lee MH, Lee YJ, et al. Weak closed-loop vibrational stimulation improves the depth of slow-wave sleep and declarative memory consolidation. Sleep. 2021;44. doi:10.1093/sleep/zsaa285
