# Supplementary material for: Investigating the impact of the years of blindness on sleep rhythms, dream patterns, and spatial abilities: The BLINDREAM protocol
Source: PLoS One. 2025 Jul 2;20(7):e0327521. doi: 10.1371/journal.pone.0327521 (PMC12221037; doi:10.1371/journal.pone.0327521)
Supplement: Attachment — Submitted filename: Response_to_Reviewers.pdf [file pone.0327521.s003.pdf]

PONE-D-25-01572
Investigating the Impact of the Years of Blindness on Sleep Rhythms, Dream Patterns, and Spatial Abilities: The BLINDREAM Protocol
PLOS ONE

Dear Dr. Vitali,

Thank you for submitting your manuscript to PLOS ONE. After careful consideration, we feel that it has merit but does not fully meet PLOS ONE's publication criteria as it currently stands. Therefore, we invite you to submit a revised version of the manuscript that addresses the points raised during the review process.
Major revisions are required. Please respond to each point raised by the reviewers, addressing all concerns regarding the theoretical background, methodological issues, and discussion.
Please submit your revised manuscript by Apr 27 2025 11:59PM. If you will need more time than this to complete your revisions, please reply to this message or contact the journal office at plosone@plos.org. When you're ready to submit your revision, log on to https://www.editorialmanager.com/pone/ and select the 'Submissions Needing Revision' folder to locate your manuscript file.

Please include the following items when submitting your revised manuscript:

- A rebuttal letter that responds to each point raised by the academic editor and reviewer(s). You should upload this letter as a separate file labeled 'Response to Reviewers'.
- A marked-up copy of your manuscript that highlights changes made to the original version. You should upload this as a separate file labeled 'Revised Manuscript with Track Changes'.
- An unmarked version of your revised paper without tracked changes. You should upload this as a separate file labeled 'Manuscript'.

If you would like to make changes to your financial disclosure, please include your updated statement in your cover letter. Guidelines for resubmitting your figure files are available below the reviewer comments at the end of this letter.

If applicable, we recommend that you deposit your laboratory protocols in protocols.io to enhance the reproducibility of your results. Protocols.io assigns your protocol its own identifier (DOI) so that it can be cited independently in the future. For instructions see: https://journals.plos.org/plosone/s/submission-guidelines#loc-laboratory-protocols. Additionally, PLOS ONE offers an option for publishing peer-reviewed Lab Protocol articles, which describe protocols hosted on protocols.io. Read more information on sharing protocols at https://plos.org/protocols?utm_medium=editorial-email&utm_source=authorletters&utm_campaign=protocols.

We look forward to receiving your revised manuscript.
Kind regards,
Serena Scarpelli
Academic Editor
PLOS ONE

We would like to thank Editor and the Reviewers for the time and effort in reviewing our manuscript and for the insightful comments proposed. We have carefully examined the points raised and addressed them in the revised version of our manuscript.

Journal Requirements:

1. When submitting your revision, we need you to address these additional requirements.
Please ensure that your manuscript meets PLOS ONE's style requirements, including those for file naming.
The PLOS ONE style templates can be found at
https://journals.plos.org/plosone/s/file?id=wjVg/PLOSOne_formatting_sample_main_body.pdf and
https://journals.plos.org/plosone/s/file?id=ba62/PLOSOne_formatting_sample_title_authors_affiliations.pdf

We reviewed PLOS ONE's style requirements and revised our manuscript accordingly. We also uploaded figures separately as individual files.

2. Thank you for stating in your Funding Statement: [This research was partially supported by the European Research Council (PI Monica Gori; Grant agreement No. 948349)].
Please provide an amended statement that declares *all* the funding or sources of support (whether external or internal to your organization) received during this study, as detailed online in our guide for authors at http://journals.plos.org/plosone/s/submit-now. Please also include the statement "There was no additional external funding received for this study." in your updated Funding Statement.
Please include your amended Funding Statement within your cover letter. We will change the online submission form on your behalf.

We added the sentence "There was no additional external funding received for this study" following the previous Funding Statement. We also provided an amended Funding Statement within your cover letter.

3. Thank you for stating the following in the Acknowledgments Section of your manuscript: [This research was partially supported by the European Research Council (PI Monica Gori; Grant agreement No. 948349)]
We note that you have provided funding information that is not currently declared in your Funding Statement. However, funding information should not appear in the Acknowledgments section or other areas of your manuscript. We will only publish funding information present in the Funding Statement section of the online submission form.
Please remove any funding-related text from the manuscript and let us know how you would like to update your Funding Statement. Currently, your Funding Statement reads as follows: "This research was partially supported by the European Research Council (PI Monica Gori; Grant agreement No. 948349). https://erc.europa.eu/homepage No the funder plays any role in the study design, data collection and analysis, decision to publish, or preparation of the manuscript."
Please include your amended statements within your cover letter; we will change the online submission form on your behalf.

We removed any funding-related text from the manuscript and updated the Funding Statement as follow "This research was partially supported by the European Research Council (PI Monica Gori; Grant agreement No. 948349). https://erc.europa.eu/homepage No the funder plays any role in the study design, data collection and analysis, decision to publish, or preparation of the manuscript. There was no additional external funding received for this study."
We also provided an amended Funding Statement within your cover letter.

4. When completing the data availability statement of the submission form, you indicated that you will make your data available on acceptance. We strongly recommend all authors decide on a data sharing plan before acceptance, as the process can be lengthy and hold up publication timelines. Please note that, though access restrictions are acceptable now, your entire data will need to be made freely accessible if your manuscript is accepted for publication. This policy applies to all data except

where public deposition would breach compliance with the protocol approved by your research ethics board. If you are unable to adhere to our open data policy, please kindly revise your statement to explain your reasoning and we will seek the editor's input on an exemption. Please be assured that, once you have provided your new statement, the assessment of your exemption will not hold up the peer review process.

This manuscript refers to a study protocol that is already publicly available on ClinicalTrials.gov (ID: NCT06631807). At this stage, no data is yet available, as the study is still ongoing. Once data collection is completed and the data has been processed and analyzed, we will make the full dataset freely available through Zenodo, in line with PLOS ONE's open data policy.

5. Please update your submission to use the PLOS LaTeX template. The template and more information on our requirements for LaTeX submissions can be found at http://journals.plos.org/plosone/s/latex.

We prepared the manuscript using a Word file in accordance with PLOS ONE's style requirements. We will provide a version using the PLOS LaTeX template if required for the final submission.

Reviewers' comments:

Reviewer #1

The research protocol aims to investigate the impact of blindness on sleep processes and analyze how sleep processes, dream content can affect spatial abilities.
The protocol is very interested and promising; however, it has some critical issues:

The Reviewer correctly summarized the main aim of the protocol. We thank you very much for your precious feedback. We are convinced that, following your suggestions, we could address all the points you raised, clarify all the aspects you highlighted, and improve the scientific rigor of the manuscript. Below is a point-by-point response to each question raised. When we specified the Lines we referred to the track changes version of the manuscript.

1) The introduction should be implemented with a more precise theoretical background consistent with the protocol assumptions. In particular, I suggest:
1.1) Describing the types of alterations in micro- and macrostructural sleep patterns observed in blind people and in those with rhythm disturbances (lines 56-59).

Thank you for your suggestion. We have provided a more detailed description of the types of alterations in micro- and macrostructural sleep patterns in blind people and how circadian rhythm can influence them, see Lines 64-75.

1.2) Describing how spatial perception is characterized in blind people and explaining how the results of Lockley and colleagues' 2008 study inform the hypotheses of the present experimental protocol (lines 65-70). I suggest a meta-analysis to expand the literature background. Bleau, M., Paré, S., Chebat, D. R., Kupers, R., Nemargut, J. P., & Ptito, M. (2022). Neural substrates of spatial processing and navigation in blindness: An activation likelihood estimation meta-analysis. Frontiers in neuroscience, 16, 1010354. https://doi.org/10.3389/fnins.2022.1010354

Thank you for your suggestion. We moved part of the Discussion describing how spatial perception is characterized in blind people in the Introduction, as suggested by Reviewer 2 (Lines 88-104). We also explained the results found by Lockley and colleagues' 2008, see Lines 80-85. Finally, we also added the interesting meta-analysis suggested.

1.3) Expand on the section from lines 80 to 83. In particular, I suggest clarifying what is meant by: 'Can the varied processing of dream content influence how sensory information is processed during our waking experiences, significantly affecting long-term performance, such as spatial abilities?' Additionally, please indicate the relevant theory. Are you also referring to mnemonic consolidation processes?"
The current literature on dreaming suggests that waking experiences can be incorporated into dream content and reprocessed. Some studies have observed that sensory stimulation in sleep affects dream content. Additionally, some theories speculate that dreaming may play a role in sleep-dependent memory consolidation processes.
The following are some relevant reference articles.
-Wamsley EJ. Dreaming and offline memory consolidation. Curr Neurol Neurosci Rep. 2014 Mar;14(3):433. doi: 10.1007/s11910-013-0433-5. PMID: 24477388; PMCID: PMC4704085
-Scarpelli S, Alfonsi V, Gorgoni M, De Gennaro L. What about dreams? State of the art and open questions. J Sleep Res. 2022 Aug;31(4):e13609. doi: 10.1111/jsr.13609. Epub 2022 Apr 13. PMID: 35417930; PMCID: PMC9539486.
-Bloxham, A., & Horton, C. L. (2024). Enhancing and advancing the understanding and study of

dreaming and memory consolidation: Reflections, challenges, theoretical clarity, and methodological considerations. Consciousness and cognition, 123, 103719.
https://doi.org/10.1016/j.concog.2024.103719

Thank you for your suggestion. We expand the section on dreams in the Introduction part, clarifying also the relevant theory as suggested, see Lines 117-129.

2) Method.
Inclusion and exclusion criteria
2.1) In lines 152-153, when discussing medical conditions, are you also referring to other sleep disorders? If so, please specify.

We are generally asked if they suffered of clinically significant and uncontrolled medical in general, also including OSAS or already diagnosed sleep disorders. We specified it.

Screening and Recruitment
2.2) For screening and recruitment, specify whether test batteries/interviews are administered to participants and which ones.

For screening and recruitment, we only evaluated during an interview the inclusion and exclusion criteria specified in the previous session. We tried to better specify it.

Design and Procedures:
2.3) At line 188 which task are you referring to? In case it is not clear how the information collection is not reduced. How do you restructure the protocol? Are PSG and actigraphy recorded simultaneously, or is one less day of actigraphy recorded?

We considered restructuring the protocol to collect both PSG and actigraphy data simultaneously during the first night. We specified it in the manuscript. However, we decided not to adopt this as the baseline approach in order to avoid overwhelming participants with too much information on the first day. However, if participants demonstrate confidence and find the procedures easy to follow, we may offer this option as it shortens the overall protocol duration and is easier to integrate into routine clinical practice.

Visit 2
2.4) Explain the instructions for dream collection. Are dreams collected in the morning within 15 minutes of waking up?
2.5) During Visit 2 why are sleep diaries not also provided to collect subjective information?

Thank you for your suggestions. We added more details about how we collect dream information. We also specify that we collect subjective information about sleep. We do not provide any additional material to participants, but we ask them to report their bed and wake times, the quality of their sleep the previous night, any nighttime awakenings, and any naps taken during the day, see Lines 291-300.

Date analysis
Hypothesis
2.6) Regarding hypotheses, it is necessary to specify what kind of spatial ability is tested in your protocol. Additionally, some hypotheses are unclear:
2.6.1) What do you mean that circadian desynchronization affects sleep structure? Are you referring

in terms of micro- and macrostructure? When you talk about "other factors" in line 285, what factors are you referring to?

As you correctly pointed out, we refer to sleep structure in terms of micro- and macrostructure. Moreover, as now specified in the Introduction, it remains unclear whether changes in sleep macro- and microstructure are a direct result of blindness or a consequence of circadian rhythm desynchronization. While accounting for circadian misalignment in analyses may appear to eliminate macro- and microstructural alterations, differences in underlying thalamo-cortical and cortico-cortical networks could still be reflected in sleep patterns. This is what we refer to as "other factors." These points have been clarified in the manuscript, see Lines 357-362.

2.6.2) Hypothesis H2.2 suggests that there may be a correlation between spatial performance and macro- and micro-structure if circadian desynchronization is not the only factor impacting night sleep in the blind. However, so formulated it is not properly correct. it is possible that both desynchronization and other sleep alterations may affect performance tasks.

Thank you for pointing out this imprecision. We agree with your comment. Indeed, as presented in H.1.2, we hypothesized that circadian desynchronization has an effect. What we aim to hypothesize in H.2.2 is that, even in the absence of circadian misalignment, differences in sleep micro- and macrostructure may still be observed due to mechanisms linked to changes in thalamo-cortical and cortico-cortical networks in the absence of vision. We have attempted to clarify this in the manuscript.

2.6.3) Hypothesis H3.1 is not entirely accurate if you are referring to dream frequency. The literature does not suggest that dream frequency directly impacts performance on specific tasks. Instead, it indicates that waking elements might be incorporated into dreams and reprocessed, but the role in memory processes is not yet clear.
Hypothesis H3.2 would be more accurate if tasks were administered before and after a night of sleep to assess whether task incorporations correlate with improved performance the following morning.

About Hypothesis H3.1, some studies have shown differences in certain cognitive performances, such as creativity, attention, and visuomotor tasks, between high dream recallers (those who recall dreams more than three times per week) and low dream recallers (Dumel et al., 2015; Ruby et al., 2022; Vallat et al., 2022), as well as some differences in the resting brain activity (Eichenlaub et al., 2014a; 2014b). The underlying mechanism is surely that dreaming may allow for greater reprocessing of information acquired during wakefulness, which in turn may influence the development of broader cognitive functions. Based on this evidence, we formulated Hypothesis H3.1. We have now clarified this point in the manuscript, see Lines 369-375.
Here, the references:

- **Dumel, G., Carr, M., Marquis, L.-P., Blanchette-Carrière, C., Paquette, T. and Nielsen, T. (2015), Infrequent dream recall associated with low performance but high overnight improvement on mirror-tracing. J Sleep Res, 24: 372-382. https://doi.org/10.1111/jsr.12286**
- **Vallat R, Türker B, Nicolas A, Ruby P. High Dream Recall Frequency is Associated with Increased Creativity and Default Mode Network Connectivity. *Nat Sci Sleep*. 2022;14:265-275 https://doi.org/10.2147/NSS.S342137**
- **Perrine Ruby, Rémy Masson, Benoit Chatard, Roxane Hoyer, Laure Bottemanne, Raphael Vallat, Aurélie Bidet-Caulet, High dream recall frequency is associated with an increase of both bottom-up and top-down attentional processes, *Cerebral Cortex*, Volume 32, Issue 17, 1 September 2022, Pages 3752–3762, https://doi.org/10.1093/cercor/bhab445**
- **Jean-Baptiste Eichenlaub, Olivier Bertrand, Dominique Morlet, Perrine Ruby, Brain Reactivity Differentiates Subjects with High and Low Dream Recall Frequencies during Both Sleep and Wakefulness, *Cerebral Cortex*, Volume 24, Issue 5, May 2014, Pages 1206–1215, https://doi.org/10.1093/cercor/bhs388**

- **Eichenlaub, JB., Nicolas, A., Daltrozzo, J. *et al.* Resting Brain Activity Varies with Dream Recall Frequency Between Subjects. *Neuropsychopharmacol* 39, 1594–1602 (2014). https://doi.org/10.1038/npp.2014.6**

About Hypothesis H3.2, we agree that a task-sleep-task design experiment could be more appropriate, and future studies will certainly move in that direction. However, to avoid further increasing the burden on participants within this protocol, we chose not to include pre- and post-sleep task assessments. However, we have revised the manuscript by replacing the term *predict*, which would indeed be inappropriate given our current design, with *association*. Our aim is to explore whether sensory content in dreams, potentially reflecting the reprocessing of daytime experiences, is generally associated with performance on specific tasks.

Statistical analysis
2.7) Given the amount of collected measures, I suggest implementing a dedicated paragraph "data analysis" to describe the computation of different variables. For example:
(a) PSG data: How will sleep scoring be conducted, and which variables will be extracted? Will spectral analyses be performed? For spindle and slow wave analyses, will a detection be performed and what parameters will be used?
(b) Regarding actigraphic measures, how will they be computed and what measures will be extracted?
(c) Melatonin measures: What analysis processes will be used?
(d) What measures will be used for performance tasks?
(e) What kind of scoring is done for dreams?
(f) Questionnaire measures: Which measures will be included in the statistical analyses? For example, will the PSQI global score or its subcomponents be used?

Thank you for your suggestion. We added the paragraph "Data Analysis" showing the different variables that will be considered during the analysis for each questionnaire, assessing device and behavioral task, see Lines 385-433.

2.8) Following this, I then suggest restructuring the statistical analyses section to specify the analyses that will be carried out for the different hypotheses, indicating for each analysis the variables considered.
We have tried to better specify the variables considered in each analysis, while avoiding redundancy with the paragraph above.

3) References
In the text some references is not numbered: For example: line 68, lines 271-272, lines 339-340, line 342.

Thank you. We have addressed this point.

Reviewer #2

Dear Authors,
Thank you for the opportunity to review your manuscript. Overall, I find the research questions to be interesting, clearly defined, and well-grounded in theory. The study protocol is well-designed and methodologically sound. I have only minor comments that may help improve the reporting of the protocol in the manuscript. Please, see the attachment for detailed comments.

Thank you very much for your time in reviewing our manuscript and positive feedback on our manuscript. We carefully addressed all the comments and suggestions provided to improve the clarity and quality of our protocol. When we specified the Lines we referred to the track changes version of the manuscript.

1. Recruitment Process:
Please clarify why certain eligibility criteria are not checked before scheduling the first visit. Would this not be part of the recruitment process?

Thank you for your comment. This referred to the possibility that a participant might be recruited during routine clinical practice and, therefore, already have a scheduled appointment for the PSG. However, since this scenario has not yet occurred in practice and we understand that the sentence may cause confusion, we have decided to remove it from the manuscript.

2. Terminology and Wording Adjustments:
2.1 Consider replacing 'quantity' with a more precise term like 'architecture', as you are measuring more than just sleep duration.
2.2 The term 'protocol' may be more appropriate than 'sleep measurements' since your study includes questionnaires as well.
2.3 'Dream quality' is somewhat ambiguous; please clarify what is meant by this term.

Thank you for the suggestions. We have updated the wording as recommended, as the proposed terms are more appropriate. We removed the ambiguous "Dream quality" and substituted it with the generic expression "dream-related aspects".
We also followed the suggestions provided in the PDF file.

3. Study Procedure:
3.1 It would be helpful to specify whether participants arrive at the hospital in the evening or at another time.
3.2 If the fourth sample coincides with their typical sleep time, does this mean they must delay sleep for the fifth sample? Please clarify the instructions given to participants.
3.3 If participants arrive in the morning, please explicitly state this in the manuscript.

Thank you for the suggestions.
To address points 3.1 and 3.3 we specified the time in which the visits will take place.
About point 3.2., for the fifth sample, participants are free to choose whether to stay awake one hour longer that evening or to go to sleep at their usual time and set an alarm to wake up and collect the sample. Since this occurs on a single night, it does not alter the participant's melatonin profile. We specified it at Lines 283-284.

4. Hypotheses:
In the hypotheses section, rephrase them as declarative statements rather than questions. For example,

instead of posing a question, state that 'Visual deprivation causes a desynchronization of the circadian rhythm in...'

Thank you for the suggestions. We rephrase them as declarative statements.

5. Discussion:
The discussion section seems to contain mostly the same information as in the introduction, which doesn't seem that helpful to me.
A lot of the discussion is repetition from the Introduction. I don't know how much sense it makes to write a 'second introduction'. The limitations at the end are great though and that is crucial for the discussion section at this stage of your project. I think most of the discussion fits in the Introduction.

Thank you for the suggestions. We have moved most of the content originally presented in the Discussion section to the Introduction, retaining only the study's aim, potential applications of the results, and limitations in the Discussion, as is typical in protocol studies.

Minor revisions improve the reporting of the protocol the manuscript's clarity and precision. I appreciate the effort that has gone into this study and believe it contributes valuable insights to the field. I look forward to seeing the final version.

Thank you very much for your valuable contribution and effort in improving this work.
